# A comparison between two lingual orthodontic brackets in terms of speech performance and patients' acceptance in correcting Class II, Division 1 malocclusion: a randomized controlled trial

**DOI:** 10.1590/2177-6709.21.4.080-088.oar

**Published:** 2016

**Authors:** Samiha Haj-Younis, Tarek Z. Khattab, Mohammad Y. Hajeer, Hassan Farah

**Affiliations:** 1 Master's student, University of Hama, School of Dentistry, Orthodontic Department, Hama, Syria.; 2 Clinical Lecturer and Demonstrator, University of Hama, School of Dentistry, Orthodontic Department, Hama, Syria.; 3 Associate Professor, Damascus University, School of Dentistry, Orthodontic Department, Damascus, Syria.; 4 Associate Professor, University of Hama, Head of the Orthodontic Department, School of Dentistry, Hama, Syria.

**Keywords:** Lingual Orthodontics, Speech, Chewing, Oral comfort.

## Abstract

**Objective:**

To compare speech performance and levels of oral impairment between two types of lingual brackets.

**Methods:**

A parallel-group randomized controlled trial was carried out on patients with Class II, Division 1 malocclusion treated at the University of Hama School of Dentistry in Hama, Syria. A total of 46 participants (mean age: 22.3 ± 2.3 years) with maxillary dentoalveolar protrusion were randomly distributed into two groups with 23 patients each (1:1 allocation ratio). Either STb (Ormco) or 7^th^ Generation (Ormco) lingual brackets were applied. Fricative sound/s/ spectrograms were analyzed directly before intervention (T_0_), one week following premolar extraction prior to bracket placement (T_1_), within 24 hours of bracket bonding (T_2_), one month after (T_3_), and three months after (T_4_) bracket placement. Patients′ acceptance was assessed by means of standardized questionnaires.

**Results:**

After bracket placement, significant deterioration in articulation was recorded at all assessment times in the 7^th^ Generation group, and up to T_3_ in the STb group. Significant intergroup differences were detected at T_2_ and T_3_. No statistically significant differences were found between the two groups in reported tongue irritation levels, whereas chewing difficulty was significantly higher in the 7^th^ Generation group one month after bracket placement.

**Conclusions:**

7^th^ Generation brackets have more interaction with sound production than STb ones. Although patients in both groups complained of some degree of oral impairment, STb appliances appeared to be more comfortable than the 7^th^ Generation ones, particularly within the first month of treatment.

## RESUMO

**Objetivo:**

comparar dois tipos de braquetes linguais, em termos de influência na fala e comprometimento da dicção.

**Métodos:**

foi realizado um estudo clínico randomizado com grupos paralelos, em pacientes portadores de má oclusão de Classe II, divisão 1, tratados na Faculdade de Odontologia da *University of Hama*, em Hama, na Síria. No total, 46 participantes (idade média de 22,3 ± 2,3 anos), com protrusão dentoalveolar maxilar foram aleatoriamente distribuídos em dois grupos com 23 pacientes cada (coeficiente de alocação = 1:1). Foram utilizados braquetes linguais STb (Ormco) ou braquetes linguais 7^th^ Generation (Ormco). Foram analisados espectrogramas do som fricativo /s/ imediatamente antes da intervenção (T_0_), uma semana após a extração dos pré-molares previamente à colagem dos braquetes (T_1_), após 24 horas da colagem dos braquetes (T_2_), um mês depois (T_3_) e três meses depois (T_4_) da colagem dos braquetes. A aceitação dos pacientes foi avaliada por meio de questionários padronizados.

**Resultados:**

após a inserção dos braquetes, registrou-se deterioração significativa da dicção, em todos os intervalos de avaliação, no grupo com os braquetes 7^th^ Generation; e até o intervalo T_3_, no grupo com braquetes STb. Foram identificadas diferenças intergrupos significativas em T_2_ e T_3_. Não foram encontradas diferenças estatisticamente significativas entre os dois grupos quanto aos níveis de irritação lingual relatados, ao passo que as dificuldades na mastigação foram significativamente maiores no grupo com braquetes 7^th^ Generation um mês após serem inseridos.

**Conclusões:**

os braquetes 7^th^ Generation apresentam maior interferência na dicção do que os braquetes STb. Embora os pacientes dos dois grupos tenham reclamado de um certo grau de comprometimento da dicção, os braquetes STb parecem ser mais confortáveis do que os braquetes 7^th^ Generation, principalmente durante o primeiro mês de tratamento.

## INTRODUCTION

Recently, the number of adults who demand orthodontic treatment is increasing and even those with high motivation are likely to have some concern about the appearance of orthodontic appliances.^1^ The lingual orthodontic technique gave an ultimate solution for patients who do not want their appliances to be shown.^2^ ^-^ ^6^ However, placement of orthodontic brackets on the lingual surfaces of teeth causes changes in their morphology, which results in articulation problems, chewing difficulties, tongue irritation and other impairments.^7^ ^-^ ^10^

Several studies have investigated patients' attitude and oral discomfort after lingual appliance placement.^8^ ^,^ ^10^ ^,^ ^11^ ^,^ ^12^ Some of them have compared lingual appliances with labial ones regarding sound production and oral discomfort,^13^ ^,^ ^14^ ^,^ ^15^ whereas another has compared different laboratory procedures and concluded that thinner appliances would enhance patients' adaptation to lingual brackets.^16^ However, few studies have focused on the effect of bracket type on oral impairment and several speech assessments have been performed semi-objectively and subjectively.^17^ ^,^ ^18^ Although acoustic analysis has been previously employed to assess speech performance in patients with specific types of lingual brackets,^12^ ^,^ ^15^ to date, there is no trial conducted to compare objectively different types of lingual brackets. 

Assessment of speech performance may differ from one language to another,^14^ and it seems that there is only one published paper comparing speech impairment between lingual and labial brackets in patients speaking the Arabic language as their native language.^15^

The current randomized controlled trial aimed to compare (1) speech performance by means of auditive analysis and (2) levels of oral impairment between two types of lingual brackets. The null hypothesis was: there are no statistically significant differences in speech performance and in levels of oral impairment between patients using either STb or 7^th^ Generation brackets.

## MATERIAL AND METHODS

### Trial design

This study was designed as a parallel-group randomized controlled trial with a 1:1 allocation ratio. No changes to the methods after trial commencement occurred.

### Participants, eligibility criteria and settings 

**Figure 1:**
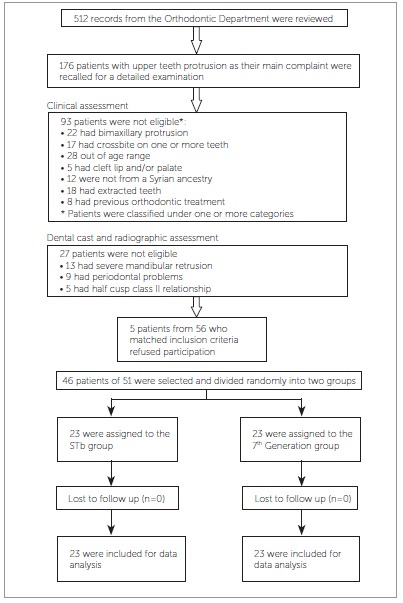
Participants flow chart.

This research project was approved by University of Hama School of Dentistry (UHDS) Ethics Committee and was funded by the University of Hama Postgraduate Research Budget. Records of 512 patients from the waiting list of the Orthodontic Department at UHDS (from January 2012 to January 2013) were reviewed. After clinical, dental cast and radiographic assessment, only 56 patients accurately met the following inclusion criteria: (1) Class II, Division 1 malocclusion; (2) age range of 15 to 30 years; (3) presence of all permanent teeth, except for third molars; (4) first language of participants was Arabic; (5) no previous orthodontic treatment; (6) no anterior crossbite; and (7) no craniofacial syndromes, cleft lip and/or palate (soft and/or hard), or history of speech and hearing disorders. A total of 51 out of 56 subjects gave their informed consent after receiving an explanation about clinical trial design orally and in a written format. A total of 46 patients (mean age: 22.3 ± 2.3 years) were randomly selected to construct the primary sample. The other five patients who gave their informed consent, but were not selected in the random selection, were treated at UHDS Orthodontic Department by other MSc students under the direct supervision of a professor (Fig 1).

### Interventions

Patients included in the study were randomly divided into two groups. Randomization was performed by means of Minitab^TM^ v.16 and conducted by one of the coauthors. A random number list was created with an allocation ratio of 1:1. Allocation procedure was concealed from the main researcher. Participants were not aware of the type of lingual appliance they received. This blinding was made to reduce the possibility of knowing to which group they belonged.

The STb group consisted of 23 patients (14 females, 9 males; mean age: 22.7 ± 2.4 years) who were treated with STb lingual brackets (Ormco Corporation, Glendora, CA, USA). The 7^th^ Generation group consisted of 23 patients (12 females, 11 males; mean age: 22.1 ± 1.9 years) who were treated with 7^th^ Generation lingual brackets (Ormco Corporation, Glendora, CA, USA).

**Figure 2:**
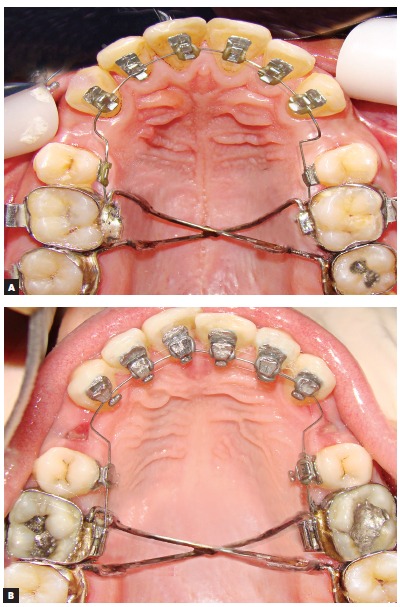
Lingual brackets used in both the STb group (A) and the 7^th^ Generation group (B).

Lingual brackets in both groups were indirectly bonded in the upper arch only by means oft he Hiro^TM^ System.^19^ Modified chromosome arches were inserted for all patients in both groups as anchorage units to prevent molar rotation, followed by first premolars extraction (Fig 2). 

### Outcomes (primary and secondary) and changes after trial commencement

#### Auditive analysis

**Figure 3:**
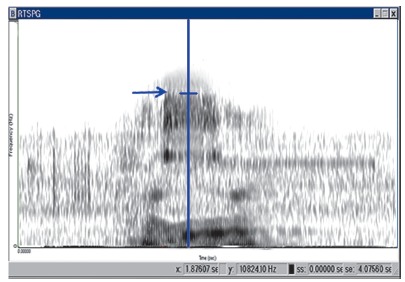
Spectrogram of the Arabic word "*Hassan*"; the arrow indicates the upper boundary frequency (UBF) of the /s/ sound.

Patients were asked to read the word "*Hassan*'' aloud in an anechoic quiet room,with digital recording achieved in standardized conditions. The target words were then sampled into a computer and the frequency of the /s/ sound was measured by means of the Kay Elemetrics CSL 3150b system (Kay Elemectrics, Pine Brook, NY, USA). This method allowed representing and recording the frequency and duration of the speech signal.^12^ ^,^ ^15^ A spectrogram was used to analyze the upper boundary frequency (UBF) of the fricative sound /s/ which was defined as the maximum frequency of the band width of the fricative sound, represented in the spectrogram as the range of maximum grayness (Fig 3). Each sound file was given a number which refers to patient's name and appliance type. A numeric list for all sound files was created by one of the coauthors, whereas analysis of spectrograms was blindly conducted by the main researcher who did not have that list. Speech performance was evaluated directly before intervention (T_0_), one week after premolar extraction and before bracket placement (T_1_), immediately following bracket placement (T_2_), one month later (± three days) (T_3_), and three months later (± one week) (T_4_). 

#### Subjective evaluation of oral comfort and patients' acceptance

At the same time points (T_0_, T_1_, T_2_, T_3_ and T_4_), each patient completed a standardized questionnaire consisting of six questions. Q1: ''Do you feel that your articulation has changed?''; Q2: ''Has a change in your articulation been noticed in your social environment?''; Q3: ''Do you avoid specific types of conversation (e.g. on the phone)?''; Q4: ''Have you noticed sores, reddening or lesion on your tongue?''; Q5: ''Do you have a sense of your tongue space being restricted?''; and Q6: ''Do you have difficulty chewing?''. Each question had four-point Likert scale possible answers: ''No, not at all'' (score 1); ''Slightly'' (score 2); ''Yes, with reserve'' (score 3); and ''Yes, indeed, I can confirm this without reserve'' (score 4).

Blindness was applied during data collection. Both the additive analysis and the questionnaire analysis were totally blinded from the main researcher.

### Sample size calculation

The following assumptions were used to calculate the required sample size using Minitab^TM^16 (Minitab Inc, State College, Pa, USA): (1) the smallest difference requiring detection in the UBF of /s/ sound was 400 Hz; (2) the significance level of two-sided tests was set at 0.05; (3) the statistical power was set at 80%; (4) standard deviation (SD) of the UBF of /s/ sound was found to be 471 Hz in a previous study^15^; (5) the intended inferential statistical approach was two-sample t-tests. Calculation revealed that a sample size of 23 patients was required for each group.

### Interim analyses and stopping guidelines

No interim analyses were applied and no stopping guidelines were employed in this trial.

### Statistical analysis

Statistical analysis was conducted by means of Minitab^TM^16 (Minitab Inc, State College, Pa, USA). Anderson-Darling normality tests were used to evaluate the distribution of collected data. Paired sample t-tests (for normally distributed variables) or Wilcoxon matched-pairs signed-rank tests (when normal distribution assumption was violated) were applied to evaluate intragroup changes. Two-sample t-tests (for normally distributed variables) or Mann-Whitney U-tests (for non-normally distributed variables) were employed to examine intergroup differences. Alpha (α) was set at 0.05.

## RESULTS

### Participant flow

A CONSORT flow diagram of participants' recruitment, follow-up and entry into data analysis is given in Figure 1. All patients in both groups have completed this study untill the end and no withdrawal or exclusion was recorded. Therefore, 46 patients were included in the analysis.

### Error of the method

Twenty sound files from both groups in all time points were randomly selected by means of computer-generated random numbers. These files were then re-analyzed for detecting the UPF of the fricative /s/ sound. Paired t-test was employed to investigate the systematic error by comparing the two sets of data. No significant differences were found.

### Auditive analysis

All patients in both groups recorded negligible and insignificant deteriorations in their articulation one week after extraction and before bracket placement (Table 1). In the STb group and before bracket placement (T_1_), the mean UBF of the /s/ sound was 12866 Hz (Table 1). At T_2_, a highly significant drop was observed (*p* < 0.001), with a mean value of 10963 Hz. The mean UBF value arose to 12507 Hz at T_3_, but still significantly differed from the record at T_1_. At T_4_, the mean UBF value increased again to reach 12717 Hz, the difference between T_4_ and T_1_ was insignificant.

**Table 1:** Speech evaluation by auditive analysis of spectrograms.

UBF (Hz)	STb group (n = 23)			7^th^ Generation group (n = 23)			*p*-value between the two groups
	Mean	SD	*p*-value	Mean	SD	*p*-value	
T_0_	12921	344		13042	468		
T_1_	12866	369	vs. T_0_ = 0.602	12987	479	vs. T_0_ = 0.697	
T_2_	10963	709	vs. T_1_ < 0.001*	9908	597	vs. T_1_ < 0.001*	
T_3_	12507	426	vs. T_1_ = 0.023*	11774	568	vs. T_1_ < 0.001*	
T_4_	12717	454	vs. T_1_ = 0.245	12647	491	vs. T_1_ = 0.036*	
Change T_2_-T_1_	- 1903	733		-3079	539		< 0.001*
Change T_3_-T_1_	- 359	629		-1213	723		= 0.003*
Change T_4_-T_1_	- 149	633		-340	730		= 0.309

T_0_: before intervention; T_1_: after extraction and before bracket placement; T_2_: immediately following bonding; T_3_: one month after; T_4_: three months post-bracket placement.*Significant difference(*p* < 0.05)

The mean UBF of the /s/ sound in the 7^th^ Generation group was 12987 Hz before bracket placement (Table 1). A highly significant drop (*p <* 0.001) was observed at T_2_, with a mean value of 9908 Hz. The mean UBF value arose significantly to 11774 Hz and to 12647 Hz at T_3_ and T_4_, respectively, but it was still significantly lower than the value recorded at T_1_. Statistically significant differences were found at T_2_ and T_3_ between the two groups, particularly with regard to the mean UBF values (Table 1). The difference detected at T_4_ was insignificant (*p =* 0.309)

### Questionnaire findings

At T_0_, all answers in relation to the six questions were identical (patients chose answer ''no, not at all"). Therefore, these data are omitted from Table 2, whereas responses at T_1_, T_2_, T_3_, and T_4_ are presented. Additionally, no significant intergroup or intragroup differences were recorded at T_1_, with the vast majority of patients in both groups answering questions with "no, not at all" (Table 2).

**Table 2:**
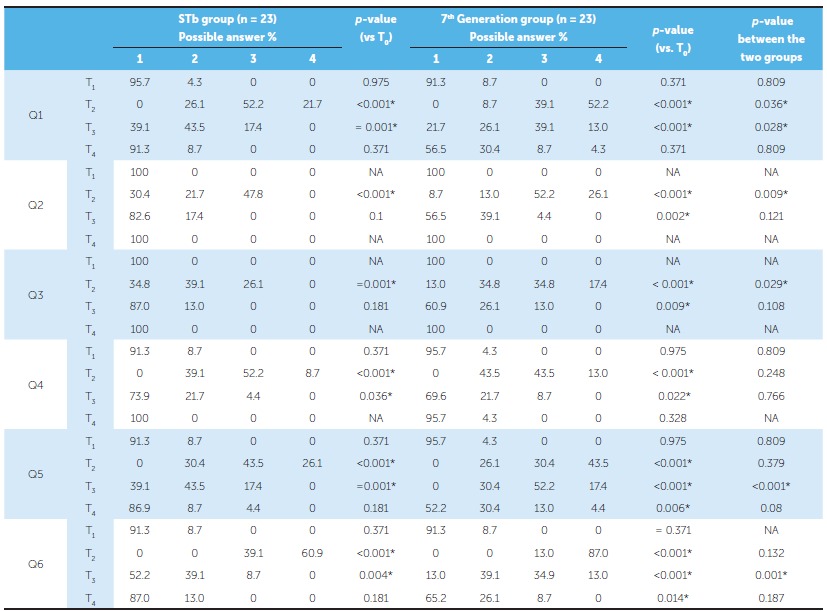
Patients' responses to the questionnaires administered at four assessment times.

Before intervention (at T_0_), all patients in both groups gave answer ''1'' for all given questions; therefore, their relevant data are not shown. T_1_ indicates one week following extraction; T_2_, immediately following bracket placement; T_3_, one month later (± three days); T_4_, three months later (± one week); NA, not applicable (ie, identical percentages of frequencies). Possible answers: 1 indicates ''No, not at all''; 2, ''slightly''; 3, ''Yes, to some degree''; and 4, ''Yes, indeed, I can confirm this.''. *Significant difference (*p* < 0.05).

### Perception of articulation change (Question 1) 

Patients in both groups noticed a significant deterioration in their articulation immediately following bracket placement (T_2_) and one month later (T_3_), with observing significant intergroup differences (*p =* 0.036 at T_2_, *p =* 0.028 at T_3_). At T_4_, moderate and severe grades of speech impairment were not recorded in the STb group, whereas they were slightly noticed in the 7^th^ Generation group, with insignificant differences between the two groups.

### Observation of articulation change by surrounding people (Question 2)

At T_2_, 47.8% of patients in the STb group had moderate noticeable speech changes as reflected by others, whereas higher degrees of social environment observation were reported by patients in the 7^th^ Generation group (52.2%). A significant intergroup difference was recorded at T_2_ (*p =* 0.009), but at T_3_ and T_4_, there were no significant differences between the two groups.

### Avoidance of some types of conversations (Question 3)

Only at T_2_ patients of both groups reported significant differences (*p* = 0.029) in their assessments about avoidance of some types of conversations.

### Irritation of the tongue (Question 4)

All patients in both groups suffered from some degree of tongue irritation at T_2_ with significant differences from the records taken at T_1_ (*p* < 0.001). All patients in the STb group and the majority of them (95.7%) in the 7^th^ Generation group reported no soreness or irritation at T_4_. No intergroup differences were recorded at all assessment times.

### Restriction of tongue space (Question 5) 

Most patients in both groups reported a moderate to severe tongue space restriction immediately after bracket placement (T_2_). An improvement was observed at T_3_, especially in the STb group which recorded a significant difference comparing with the 7^th^ Generation group (*p* < 0.001).

At T_4_, most patients in the STb group (86.9%) claimed having no tongue space restriction, while about half of patients in the 7^th^ Generation group recorded different degrees of tongue space restriction. However, this difference between the two groups was insignificant (*p* = 0.08) 

### Difficulty of mastication (Question 6)

Immediately after appliance placement (T_2_), all patients (100%) in both groups suffered from moderate to severe chewing problems, and no significant intergroup difference was detected (*p* = 0.132). The mastication ability improved at T_3_ when about half patients (52.2%) in the STb group reported being able to eat comfortably, whereas a significantly less improvement was observed in the 7^th^ Generation group (*p* = 0.001). Another improvement was registered at T_4_ in both groups and no significant difference was recorded.

## DISCUSSION

It seems that the current study is the first randomized controlled trial carried out to compare speech performance between two types of lingual brackets by means of digital sonography combined with subjective auditive analysis. A significant drop of the UBF of the consonant /s/ sound was recorded directly after bracket bonding (T_2_) in both groups. However, patients in the 7^th^ Generation group showed more reduction and higher degrees of impairment in comparison to those in the STb group. This finding can be explained by the difference in bracket dimensions, since the STb brackets were considerably smaller than the 7^th^ Generation ones, bucco-lingually and cervico-incisally. Therefore, less interaction with sound production was noticed. The effect of lingual appliance thickness on speech performance was studied previously by Hohoff et al^17^ who assessed articulation semi-objectively employing blinded speech professionals and subjectively using a standardized questionnaire in patients with three types of lingual appliances. The authors concluded that the thinner the appliance, the less interaction with pronunciation. 

The fricative /s/ sound is well known to be sensitive to morphological changes in maxillary incisors and it is common in most languages; therefore, it is well suited for evaluating speech performance.^20^ ^,^ ^21^ The Arabic word "*Hassan*" was evaluated previously by Khattab et al^15^ who recorded more reduction in the UBF of the /s/ sound in the lingual bracket group than what was reported in the current study. This difference can be explained by the different types of lingual brackets used. Khattab et al^15^ evaluated Stealth lingual brackets (American Orthodontics, Sheboygan, WI, USA) which had sharp hooks, causing more irritation in the tongue and affecting the production of the /s/ sound, whereas both the small dimensional STb and the rounded hooks of the 7^th^ Generation brackets, which were used in the current study, caused less irritation and thus less intervention with sound production.

Hohoff et al^12^ investigated the UBF of the fricative /s/ sound after applying 7^th^ Generation lingual brackets, and reported less dropped values than what was found in the current study. This can be attributed to variation of voice pattern while pronouncing different words in different languages: Hohoff et al^17^ analyzed the articulation of the /s/ sound in the French word "*soleil*" at the end of the sentence "*La brise et le soleil*"; whereas the stressed "s" sound in the Arabic word "*Hassan*" was evaluated in our study.

According to questionnaire findings, patients in both groups reported highly significant impairment in their articulation until one month of assessment. However, speech difficulty in the 7^th^ Generation group was significantly greater than in the STb group at T_1_ and T_2_. This finding can be explained by the different bracket size between the two groups: 7^th^ Generation appliances are considerably thicker than the STb ones, especially in the premolar region. For this reason, they had more interaction with speech production. After three months of appliance wear, a few patients still complained of some degree of speech impairment. These findings are generally in agreement with those of other studies;^11^ ^,^ ^13^ ^,^ ^14^ ^,^ ^15^ however, the differences in study design, types of brackets and the language of choice have caused variations of intensity and overall duration of oral impairment. Patients in both groups complained of different degrees of tongue space restriction which gradually disappeared with time. Nevertheless, the only significant difference between the two groups was detected one month after bracket placement when the 7^th^ Generation appliance, which is considerably thicker than the STb one, caused more narrowing of tongue space. It was confirmed previously that the thinner the lingual appliance, the less intervention with tongue space.^16^ ^,^ ^17^ ^,^ ^18^ The different findings between the objective assessment (auditive analysis) and the subjective assessment (questionnaires) was due to the difference between these two methods. The auditive analysis investigated the changes of the fricative /s/ sound only, whereas when patients were asked to assess their speech performance by means of questionnaires, they gave themselves as well as surrounding people, the perception of any noticeable change in the overall speech performance. In addition, this subjective assessment could be affected by patients' emotional circumstances.

All patients in both groups suffered from different degrees of irritation and soreness of the tongue, which disappeared at the three-month assessment. However, patients in the 7^th^ Generation group were insignificantly more often affected than those in the STb group. Stamm et al^18^ recorded a significant difference between 7^th^ Generation brackets and customized brackets (Incognito, TOP Service, Ormco) with respect to pressure sores and lesions of the tongue. Khattab et al^15^ reported higher levels of irritation after the use of lingual brackets. This difference can be attributed to the sharp hooks of the Stealth^(r)^ (American Orthodontics, Sheboygan, WI, EUA) brackets that they used in their study.

Chewing difficulties were observed in both groups, but were significantly more severe in the 7^th^ Generation group one month after bracket placement. This finding is in agreement with the results of Stamm et al^18^ who found that 7^th^ Generation brackets caused significantly more chewing impairment than that caused by customized brackets (Incognito, TOP Service, Ormco). Stealth brackets, which were investigated previously, caused more eating problems than STb and less than 7^th^ Generation brackets.^17^ This difference can be explained by the effect of bracket thickness on oral discomfort, since stealth brackets stand in the middle between STb and 7^th^ Generation brackets regarding size. Wiechmann et al^22^ reported lower levels of mastication problems when they used customized brackets (Incognito, TOP Service, Ormco), in which the resulted appliance is considerably thinner than that produced by the Hiro technique.

According to questionnaire analysis, chewing difficulty was the most severe problem caused by lingual bracket placement. These findings resemble those of Khattab et al,^15^ but do not concur with those of Caniklioglu and Ozturk^14^ and Wu et al^13^ who reported speech difficulty as the most annoying problem in patients treated with lingual brackets. Our findings also do not agree with those by Fillion ^9^ and Fritz et al^10^ who found that tongue irritation was the most serious problem with lingual appliances. This may be due to many factors, such as study design, types of brackets, laboratory procedures and the researched populations.

No harms or severe untoward effects were observed during this trial. On the other hand, there were a number of limitations in the current study. Larger sample sizes are required to investigate the effect of both age and sex which were not considered in this study. Although the current study compared two commonly used different sized lingual brackets, there is still a need for further investigation using other available lingual brackets. The generalizability of the findings of the current study might be limited, since this trial focused on a specific type of malocclusion with only two designs of lingual brackets under consideration. In addition, analysis was based on one single consonant uttered from Arabic speaking patients. An expanded auditive analysis is required in future research work.

## CONCLUSIONS

- Patients with 7^th^ Generation lingual brackets had higher degrees of speech impairment based on both auditive analysis and subjective questionnaire-based analysis.
- Both types of appliances caused soft tissue irritation and chewing difficulty, but patients with STb brackets were generally more comfortable than those with 7^th^ Generation brackets.
- Most patients in both groups reported improvements in their assessments by the fourth assessment period with gradually improvement by time.
